# Disparate effects of MMP and TIMP modulation on coronary atherosclerosis and associated myocardial fibrosis

**DOI:** 10.1038/s41598-021-02508-4

**Published:** 2021-11-30

**Authors:** Georgios Kremastiotis, Ishita Handa, Christopher Jackson, Sarah George, Jason Johnson

**Affiliations:** Laboratory of Cardiovascular Pathology, Translational Health Sciences, Bristol Medical School, Faculty of Health Sciences, University of Bristol, Level 7, Bristol Royal Infirmary, Bristol, BS2 8HW England, UK

**Keywords:** Cardiovascular diseases, Myocardial infarction, Mechanisms of disease, Proteolysis

## Abstract

Matrix metalloproteinase (MMP) activity is tightly regulated by the endogenous tissue inhibitors (TIMPs), and dysregulated activity contributes to extracellular matrix remodelling. Accordingly, MMP/TIMP balance is associated with atherosclerotic plaque progression and instability, alongside adverse post-infarction cardiac fibrosis and subsequent heart failure. Here, we demonstrate that prolonged high-fat feeding of apolipoprotein (Apo)e-deficient mice triggered the development of unstable coronary artery atherosclerosis alongside evidence of myocardial infarction and progressive sudden death. Accordingly, the contribution of select MMPs and TIMPs to the progression of both interrelated pathologies was examined in Apoe-deficient mice with concomitant deletion of Mmp7, Mmp9, Mmp12, or Timp1 and relevant wild-type controls after 36-weeks high-fat feeding. Mmp7 deficiency increased incidence of sudden death, while Mmp12 deficiency promoted survival, whereas Mmp9 or Timp1 deficiency had no effect. While all mice harboured coronary disease, atherosclerotic burden was reduced in Mmp7-deficient and Mmp12-deficient mice and increased in Timp1-deficient animals, compared to relevant controls. Significant differences in cardiac fibrosis were only observed in Mmp-7-deficient mice and Timp1-deficient animals, which was associated with reduced capillary number. Adopting therapeutic strategies in Apoe-deficient mice, TIMP-2 adenoviral-overexpression or administration (delayed or throughout) of a non-selective MMP inhibitor (RS-130830) had no effect on coronary atherosclerotic burden or cardiac fibrosis. Taken together, our findings emphasise the divergent roles of MMPs on coronary plaque progression and associated post-MI cardiac fibrosis, highlighting the need for selective therapeutic approaches to target unstable atherosclerosis alongside adverse cardiac remodelling while negating detrimental adverse effects on either pathology, with targeting of MMP-12 seeming a suitable target.

## Introduction

A wide range of cardiovascular pathologies including, but not limited to, atherosclerotic plaque progression and rupture, and ensuing adverse post-myocardial infarction (MI) remodelling are highly dependent on extracellular matrix (ECM) turnover^1^. Matrix metalloproteinases (MMPs) belong to a family of more than 25 zinc-dependent proteolytic enzymes and are the main ECM degrading proteases^2^. MMP activity is tightly regulated by the endogenous tissue inhibitors of metalloproteinases (TIMPs) that oppose to matrix degradation. An imbalance between MMP and TIMP levels results in dysregulated proteolytic activity and commonly unfavourable ECM remodelling, and is associated with coronary artery atherosclerotic plaque progression and instability, alongside adverse post-MI fibrosis and subsequent heart failure^2^.

During atherosclerotic plaque development and progression, hypercholesterolaemia is the pivotal adverse stimuli that drives macrophage foam cell formation^3^. Advanced atherosclerotic plaques are frequently categorised as stable or vulnerable, based on their composition and, consequently, their predisposition to rupture^4^. A stable atherosclerotic plaque is characterised by a thick fibrous cap, rich in vascular smooth muscle cells (VSMCs) and ECM proteins including fibrillar collagens. Heightened MMP activity has been associated with atherosclerotic plaque growth and destabilisation^5^. With increased apoptosis of macrophage foam cells alongside degradation of the fibrous cap, due to increased MMP activity, coronary atherosclerotic plaques become increasingly prone to rupture, which can trigger occlusive thrombus formation and induce an MI^4,6^. Following an MI, the wound healing process occurring in response to the ischaemic injury is characterised by inflammatory cell infiltration, namely monocytes and monocyte-derived macrophages, which accumulate and remove myocyte debris, alongside population of the infarcted tissue by activated cardiac fibroblasts, responsible for the formation of a fibrotic scar^7,8^. The structural, cellular, and extracellular changes that occur in response to such adverse events are characterised as cardiac remodelling and are crucial for the efficient repair and adaptation of the heart after ischaemia^9^. During post-MI cardiac remodelling, there is compelling evidence that a wide range of MMPs and TIMPs are regulated in a temporal and spatial manner, therefore orchestrating ECM remodelling^2,10^. However, a dysregulated MMP/TIMP balance and ensuing aberrant ECM turnover results in maladaptive structural and functional changes to the left ventricle that lead to decreased contractile function and increases the risk of developing heart failure^9,11^.

In the present study, the role of specific MMPs and TIMPs in coronary atherosclerosis-related myocardial remodelling is investigated. In particular, MMP-7, -9, and -12 and TIMP-1 and TIMP-2 were studied, based on their identified roles in the progression and destabilisation of brachiocephalic plaques within atherosclerotic Apolipoprotein E-deficient (Apoe^−/−^) mice^5,12,13^. For this purpose, chronic hypercholesterolaemic Apoe^−/−^ mice were used and the effect of specific MMP/TIMP modulations was investigated in regard to occurrence of sudden death, coronary artery atherosclerosis, cardiac fibrosis, and remodelling.

## Materials and methods

### Animals

Mice on an Apoe^−/−^ background were used, as a well-characterised and reproducible model of hypercholesterolaemia and spontaneous development of atherosclerosis, in comparison to Apoe^+/+^ mice which are not rendered hypercholesterolaemic upon high-fat feeding and are therefore considered athero-resistant^14,15^.

#### MMP/TIMP knockout studies

MMP-7^−/−^ (original background strain C57BL/6)^16^, MMP-9^−/−^ (original background strain CD1)^17^, and MMP-12^−/−^ (original background strain 129)^18^ mice were kindly provided by Dr J Mudgett (Merck Research Laboratories, Rahway, New Jersey) and Dr S Shapiro (Harvard Medical School, Boston, Massachusetts). TIMP-1^−/−^ (original background strain C57BL/6 J:129)^19^ mice were kindly provided by Dr R Lijnen (University of Leuven, Leuven, Belgium). Apoe^−/−^ mice (background strain 71% C57BL/6, 29% 129) were crossed with MMP/TIMP knockout mice to generate Apoe^−/−^/MMP-7^−/−^, Apoe^−/−^/MMP-9^−/−^, Apoe^−/−^/MMP-12^−/−^, and Apoe^−/−^/TIMP-1^−/−^ double knockout mice, as well as their corresponding age-, sex-, and strain-matched Apoe^−/−^ single knockout littermate controls. Male and female mice, aged 8 weeks, were fed a high-fat diet comprising of 21% (wt:wt) pork lard and supplemented with 0.15% (wt:wt) cholesterol (Special Diet Services, Witham, UK) for 36 weeks.

#### MMP inhibition studies

Apoe^−/−^ female mice (background strain 71% C57BL/6, 29% 129), aged 8 weeks, were fed a high-fat diet containing a broad-spectrum MMP inhibitor, RS-130830, at a concentration of 240 mg/kg of diet. RS-130830 was administered either throughout, for 36 weeks, or delayed, with treatment commencing for 20 weeks following 16 weeks of high-fat diet, in order to investigate the effect of MMP inhibition on established coronary plaques.

#### TIMP-2 over-expression study

Apoe^−/−^ male mice (background strain 71% C57BL/6, 29% 129), aged 8 weeks, were fed a high-fat diet for 18 weeks. A helper-dependent adenoviral vector expressing mouse TIMP-2 (TIMP-2 HD) or an empty adenoviral vector (empty HD) were administered, once, following 7 weeks of high-fat diet. Construction and optimisation of the TIMP-2 HD are previously described^13^.

The housing and care of the animals and all the procedures used in these studies were performed in accordance with the ethical guidelines and regulations of the University of Bristol and the UK Home Office, with all in vivo studies approved by the Animal Welfare and Ethics Review Body (AWERB), University of Bristol. Adherence to the ARRIVE guidelines (https://arriveguidelines.org) for the reporting of animal in vivo experiments was also followed.

### Termination

Animals were anaesthetised using intraperitoneal injection of sodium pentobarbitone (500 mg/kg of bodyweight), followed by exsanguination by arterial perfusion through the abdominal aorta with PBS at a constant pressure of 100 mmHg, with outflow via the incised jugular veins. Hearts were perfused-fixed and removed from each animal.

### Histology

Hearts from Apoe^−/−^/MMP-7^−/−^, Apoe^−/−^/MMP-9^−/−^, Apoe^−/−^/MMP-12^−/−^, Apoe^−/−^/TIMP-1^−/−^ and Apoe^−/−^ mice with MMP inhibition are corresponding controls (n = 10/group) and TIMP-2 HD and corresponding controls (n = 7–9/group) were used. Hearts were cut into three equal transverse pieces and subjected to histological processing, embedded in paraffin and serial 3 μm sections were cut. Successive paraffin sections were dewaxed and rehydrated before proceeding with histochemical staining. with Miller’s elastin/van Gieson (EVG) for the assessment of coronary atherosclerosis. Consequently, atherosclerotic burden and fibrosis were assessed within up to four heart cross-sections per mouse, between 30 and 50 µm apart.

### Analysis of myocardial fibrosis

To determine and assess areas of potential myocardial infarction, evidenced as myocardial fibrosis, cross-sections were histochemically stained with Masson's trichrome (Sigma, Gillingham, UK) or subjected to immunohistochemistry using fluorescein-conjugated wheat germ agglutinin (WGA) (FL-1021; Vector, Oxfordshire, UK) at 5 μg/mL. Fibrosis was quantified by measuring the Masson's trichrome-stained (blue) or fluorescein-WGA labelled (green) area using a computer-assisted image analysis system (ImageProPlus, Media Cybernetics), and expressed as area of fibrosis per myocardial cross-section.

### Immunohistochemistry

Successive 3 μm paraffin sections were dewaxed and rehydrated. Incubation with Bloxall (Vector Labs, Cambridgeshire, UK) was used to inhibit endogenous peroxidase activity followed by 20% (v/v) goat serum in PBS. For the identification of cardiomyocytes, sections were subsequently incubated with an anti-α-actinin rabbit antibody (11,313–2-AP; Proteintech, Manchester, UK) at 2 μg/mL, followed by biotinylated anti-rabbit IgG goat antibody (A-11012; Invitrogen; Fisher Scientific, Loughborough, UK) at 20 μg/mL, whereas capillaries were identified (endothelial cells), through incubation with biotin-conjugated Griffonia Simplicifolia Isolectin B4 (L2140; Sigma, Poole, UK) at 10 μg/mL in PBS. All sections were then incubated with horseradish peroxidase-labelled Extravidin™ (diluted 1:400 in 1% (w/v) BSA in PBS) and colour developed with SIGMA*FAST*™ 3,3′-Diaminobenzidine (D4293; Sigma, Poole, UK), following which nuclei were counterstained with Mayer’s haematoxylin. Positive cells were counted and expressed as a percentage of the total number of nucleated cells. A negative control was always included. In this case, the primary antibody was substituted with mouse or rat IgG of the same dilution.

For dual immunohistochemistry, sections were first incubated for 30 min at room temperature with biotin-conjugated Griffonia Simplicifolia Isolectin B4 (IB4) (L2140; Sigma, Poole, UK) at 10 μg/mL, followed by horseradish peroxidase-labelled Extravidin (diluted 1:400 in 1% (w/v) BSA in PBS) and colour developed with SIGMA*FAST*™ 3,3′-Diaminobenzidine (D4293; Sigma, Poole, UK). Sections were then washed with PBS and incubated overnight at 4ºC with a rabbit polyclonal antibody against anti-α-actinin (11,313–2-AP; Proteintech, Manchester, UK) at 0.25 μg/mL diluted in PBS. The next day sections were incubated with goat anti-rabbit biotinylated secondary antibody (E0432; Dako, High Wycombe, UK) diluted 1:200 in 1% (w/v) BSA in PBS, followed by alkaline phosphatase-labelled Extravidin (diluted 1:400 in 1% (w/v) BSA in PBS) and colour developed with SIGMA*FAST*™Fast Red TR/Naphthol AS-MX (F4648; Sigma, Poole, UK). Subsequently, nuclei were counterstained with Mayer’s haematoxylin (MHS1; Sigma, Poole, UK). The specificity of the immunolabelling was demonstrated by inclusion of a negative control using isotype-specific nonimmune serum or IgG. Images were acquired from a brightfield microscope using ImageProPlus image analysis software (Media Cybernetics). IB4 positive cells (brown colour) depicting capillaries were counted and expressed as the number of capillaries per × 10 magnification field. The number of α-actinin -positive cells (myocytes; red colour) in the same regions as examined for capillary density, were also counted. The reproducibility of assessments was tested for interobserver variation by using an unweighted κ test (κ = 0.75).

### Morphological analysis

The presence and prevalence of atherosclerotic coronary arteries was quantified within myocardial cross-sections subjected to EVG histochemical staining and expressed as percentage of atherosclerotic burden, which refers to the percentage of coronary arteries containing atherosclerotic plaques per mouse heart cross-section.

### Analysis of myocardial capillary density

Within Griffonia Simplicifolia Isolectin B4 (IB4) immunolabelled sections, the number of capillaries (IB4-positive endothelial cells) was counted in nine × 10 magnification fields using a computer-assisted image analysis system (ImageProPlus, Media Cybernetics), and expressed as capillary density per × 10 field of myocardium.

### Analysis of myocardial myocyte density

Within anti- α-actinin immunolabelled sections, the number of myocytes was counted in nine × 10 magnification fields using a computer-assisted image analysis system (ImageProPlus, Media Cybernetics), and expressed as myocyte density per × 10 field of myocardium.

### Statistical analysis

All values are presented as mean ± standard error of the mean (SEM). When comparing group means, groups were tested for normal distribution with D’Agostino-Pearson test. For normally distributed groups, an unpaired t-test, or ordinary one-way ANOVA with Tukey’s multiple comparisons post-hoc test was used. For groups that were not normally distributed, a Mann–Whitney test or Kruskal–Wallis test with Dunn’s multiple comparisons post-hoc test was used. Survival data were compared using the Kaplan–Meier method, with the log-rank (Mantel-Cox) test for significance. Statistical significance is reported as *P < 0.05, **P < 0.01, ***P < 0.001, and †*P* < 0.05 is used when non-parametric test was used.

## Results

### Effect of MMP/TIMP modulation on sudden death in Apoe^−/−^ mice

We have previously demonstrated that modulation of MMP expression and/or activity using the approaches detailed within this study affects atherosclerosis within the brachiocephalic artery of short-term (8–10 weeks) high-fat fed Apoe^−/−^ mice^5,13,20^. In addition, prolonged high-fat feeding of Apoe^−/−^ mice is associated with the development of unstable atherosclerotic plaques within the brachiocephalic artery, alongside indicators of MI and sudden death^21,22^. Accordingly, specific MMP/TIMP modulation models were developed in Apoe^−/−^ mice in order to determine their contributory role to coronary artery atherosclerosis and associated effect on myocardial fibrosis and remodelling (summarised in Supplementary Table 1).

**Figure 1:**
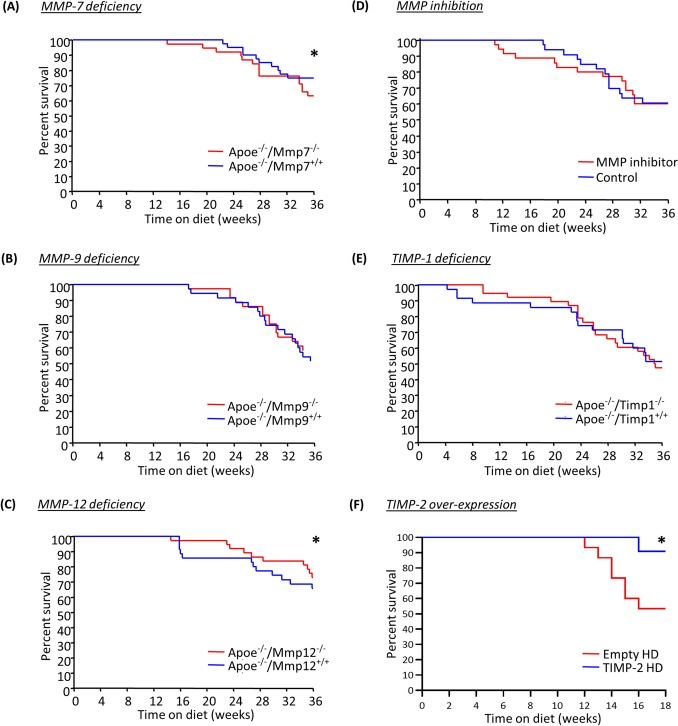
Effect of MMP/TIMP modulation on sudden death in apolipoprotein E-deficient (Apoe^-/-^) mice. (**A**–**D**) Kaplan–Meier curves of percent survival in 36-week high-fat fed Apoe^-/-^ mice also deficient for (**A**) MMP-7, (**B**) MMP-9, (**C**) MMP-12, or (**D**) TIMP-1 in comparison to their respective Apoe single knockout controls. (**E**) Kaplan–Meier curve of percent survival in 36-week high-fat fed Apoe^-/-^ mice treated with a broad-spectrum MMP inhibitor, both delayed and throughout, or without. (**F**) Kaplan–Meier curve of percent survival in 18-week high-fat fed Apoe^-/-^ mice with administration of a TIMP-2 helper-dependent (HD) adenovirus or an empty HD as control. Statistical comparisons were made using log-rank test, with *P < 0.05. In A-E, n = 36/group; in F, n = 13/group.

Apoe/MMP double knockout mice presented with divergent effects on the incidence of sudden death. MMP-7 deficiency resulted in significantly lower survival after 36 weeks of high-fat feeding (Fig. 1A). Contrastingly, MMP-12 deficiency markedly reduced the frequency of sudden death in Apoe^−/−^ mice in comparison to their wild-type controls (Fig. 1C), whereas no significant difference on survival was observed between MMP-9 double knockout and Apoe single knockout control mice (Fig. 1B). Similarly, inhibition of MMP activity with a broad-spectrum MMP inhibitor, RS-130830, or TIMP-1 deficiency had no significant effect on the prevalence of sudden death (Fig. 1D,E). Conversely, helper-dependent adenovirus-mediated TIMP-2 overexpression dramatically lowered the proportion of animals suffering sudden death and promoted survival of Apoe^−/−^ mice over the course of 18 weeks of high-fat diet (Fig. 1F).

### Evidence of unstable coronary artery atherosclerotic plaques alongside signs of myocardial infarction in Apoe^−/−^ mice

**Figure 2:**
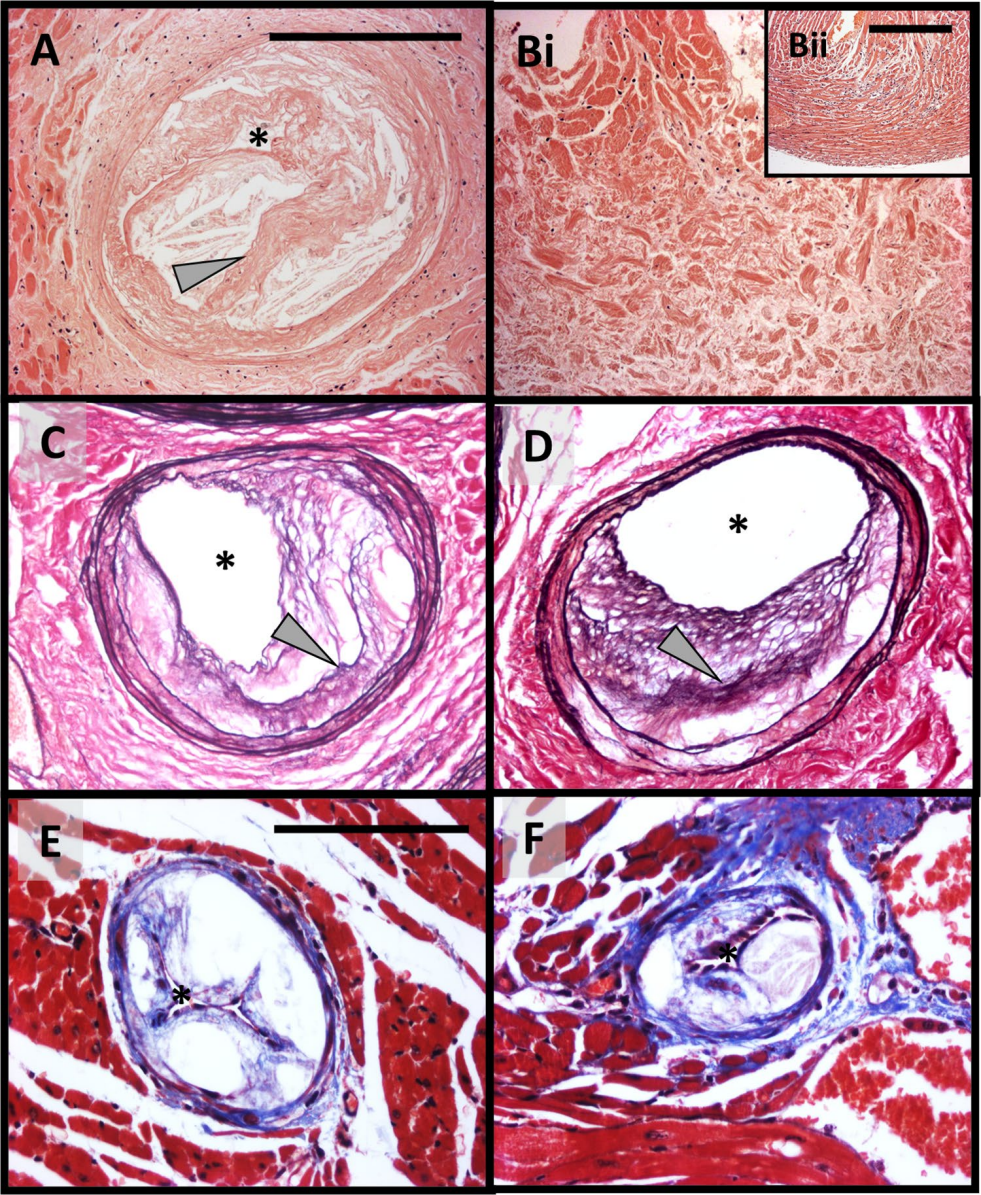
Representative atherosclerotic coronary arteries with signs of healed plaque rupture, alongside evidence of myocardial infarction. (**A** and **B**) Haematoxylin and eosin stained (**A**) proximal left anterior descending coronary artery with a large atherosclerotic lesion harbouring a healed plaque rupture (arrow), within (Bi) a heart with a large area of infarction alongside (Bii) healthy myocardium, from a 36-week high-fat fed Apoe^-/-^ mouse. (**C** and **D**) Elastin van Gieson stained proximal coronary artery lesions exhibiting healed plaque ruptures (arrows), from a 36-week high-fat fed Apoe^-/-^ mouse. (**E** and **F**) Masson trichrome stained distal coronary arteries harbouring large occlusive atherosclerotic lesions, from a 36-week high-fat fed Apoe^-/-^ mouse. Asterisk indicates lumen. Scale bar in Panel A represents 200 μm and is applicable to Panels (**A**), Bi, (**C** and **D**). Scale bar in Panel Bii represents 200 μm. Scale bar in Panel (**E**) represents 100 μm and applies to Panels (**E** and **F**).

Given the prevalence of sudden death within Apoe^−/−^ mice alongside the effects of MMP/TIMP modulation on survival rates, hearts were histologically assessed for signs of coronary atherosclerosis and MI. Left anterior descending (LAD) coronary arteries from 36-week high-fat fed Apoe^−/−^ mice regularly contained large occlusive atherosclerotic plaques with the presence of buried fibrous layers (Fig. 2A), indicative of a healed plaque rupture^23^. Interestingly, analysis of serial sections, downstream of coronary arteries with signs of healed plaque rupture, revealed areas of MI (Fig. 2Bi), as surmised through loss of myocytes alongside signs of fibrosis, in comparison to healthy myocardium (Fig. 2Bii). Established atherosclerotic plaques containing buried fibrous layers were also observed within proximal coronary arteries (Fig. 2C,D), alongside occlusive atherosclerotic lesions within distal coronary arteries (Fig. 2E,F). Collectively, these observations demonstrate that prolonged high-fat feeding of Apoe^−/−^ mice is commonly associated with the presence of unstable coronary atherosclerosis and signs of MI, supporting its role as a valuable animal model of spontaneous atherosclerosis-induced MI, and post-MI cardiac remodelling.

### Effect of MMP/TIMP modulation on coronary artery atherosclerosis in Apoe^−/−^ mice

**Figure 3:**
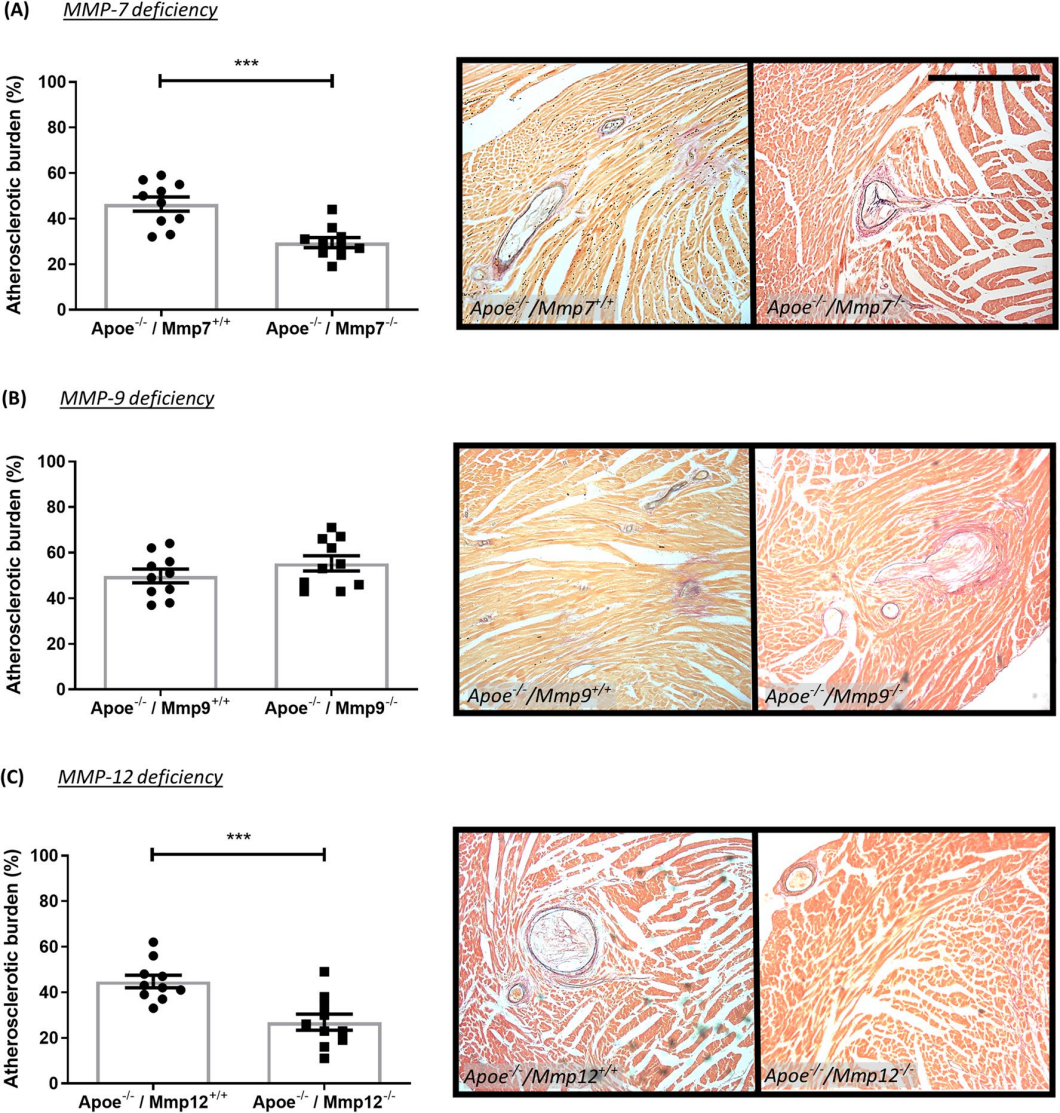
Effect of MMP modulation on coronary artery atherosclerosis in apolipoprotein E-deficient (Apoe^-/-^) mice. Quantification and representative images of coronary atherosclerotic burden as assessed in EVG-stained hearts from 36-week high-fat fed Apoe^-/-^ mice deficient for (**A**) MMP-7, (**B**) MMP-9, and (**C**) MMP-12, in comparison to their respective Apoe single knockout controls. Atherosclerotic burden (%) is presented as average plaque burden/coronary artery/mouse. Statistical significance is reported as ***P < 0.001 using unpaired Students t-test. Scale bar represents 1 mm and is applicable to all panels. In all cases, n = 10/group.

**Figure 4:**
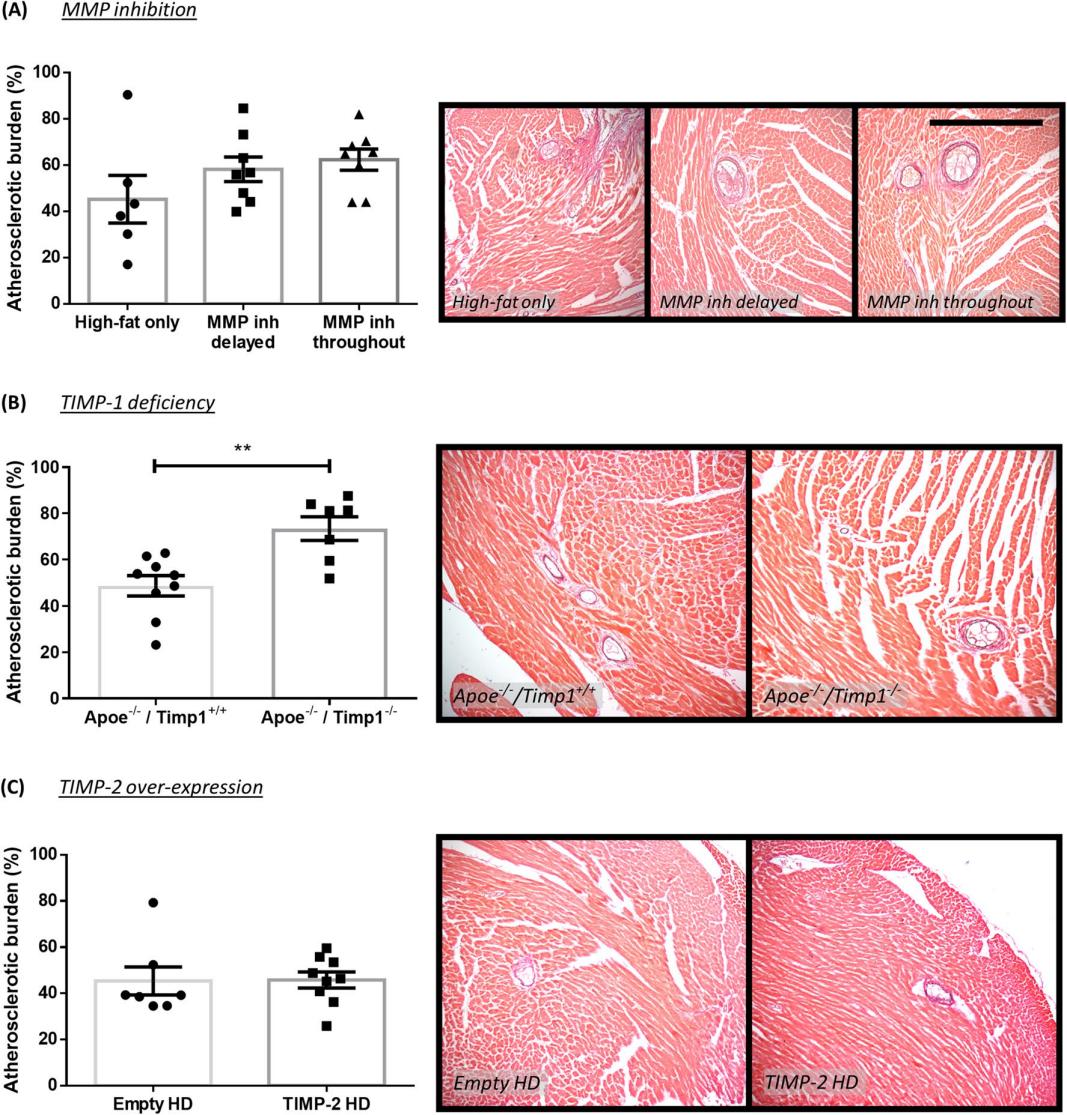
Effect of MMP inhibition and TIMP modulation on coronary artery atherosclerosis in apolipoprotein E-deficient (Apoe^-/-^) mice. Quantification and representative images of coronary atherosclerotic burden as assessed in EVG-stained hearts from (**A**) 36-week high-fat fed Apoe^-/-^ mice treated with or without a broad-spectrum MMP inhibitor, (**B**) 36-week high-fat fed Apoe^-/-^ mice deficient for TIMP-1 in comparison to Apoe single knockout controls, and (**C**) 18-week high-fat fed Apoe^-/-^ mice with administration of a TIMP-2 helper-dependent (HD) adenovirus or an empty HD as control. Atherosclerotic burden (%) is presented as average plaque burden/coronary artery/mouse. Statistical significance is reported as **P < 0.01 using unpaired Students t-test. Scale bar represents 1 mm and is applicable to all panels. In A, n = 6–8/group; in B, n = 7–9/group; in C, n = 7–9/group.

The effect of MMP/TIMP modulation on atherosclerosis development was assessed through quantification of coronary artery atherosclerotic burden. MMP-7 (36%; *P* < 0.001; Fig. 3A) and MMP-12 deficiency (40%; *P* < 0.001; Fig. 3C) resulted in significantly reduced atherosclerotic burden, compared to strain-matched Apoe single knockout controls, whereas no effect was observed with MMP-9 deficiency (Fig. 3B). Similarly, MMP inhibition (delayed or throughout) had no significant effect on atherosclerosis development (Fig. 4A). In regard to TIMP modulation, TIMP-1 deficiency significantly increased atherosclerotic burden in Apoe^−/−^ mice (1.5-fold; *P* < 0.01; Fig. 4B), whereas TIMP-2 overexpression had no effect (Fig. 4C).

Plaque progression was further assessed through the quantification of coronary arteries displaying greater than 50% stenosis. In line with the findings pertaining to atherosclerotic burden, Apoe/TIMP-1 double knockout mice showed a significant increase in plaque stenosis in comparison to strain-matched Apoe single knockout controls (1.8-fold; *P* < 0.05; Supplemental Fig. 1e). However, deficiency of MMP-7, MMP-9, or MMP-12, MMP inhibition, and TIMP-2 overexpression did not affect the proportion of coronary plaques with > 50% stenosis (Supplemental Fig. 1).

### Effect of MMP/TIMP modulation on cardiac fibrosis in atherosclerotic Apoe^−/−^ mice

**Figure 5:**
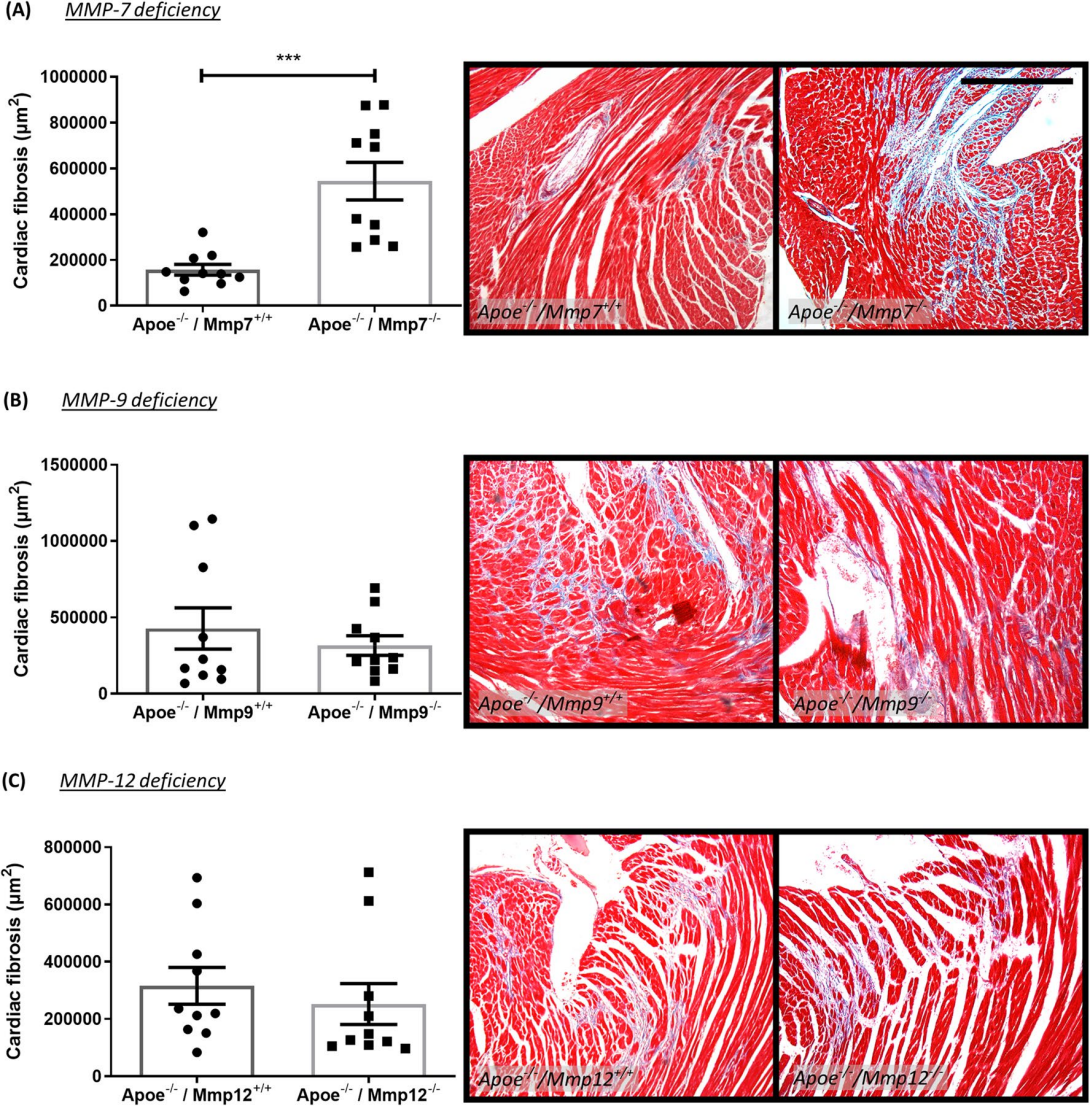
Effect of MMP modulation on cardiac fibrosis in apolipoprotein E-deficient (Apoe^-/-^) mice. Quantification and representative images of cardiac fibrosis as assessed in Masson’s trichrome-stained hearts from 36-week high-fat fed Apoe^-/-^ mice deficient for (**A**) MMP-7, (**B**) MMP-9, and (**C**) MMP-12, in comparison to their respective Apoe single knockout controls. Cardiac fibrosis is presented as average fibrillar collagen content (blue) in µm^2^/heart cross-section/mouse. Statistical significance is reported as ***P < 0.001 using unpaired Students t-test. Scale bar represents 1 mm and is applicable to all panels. In all cases, n = 10/group.

**Figure 6:**
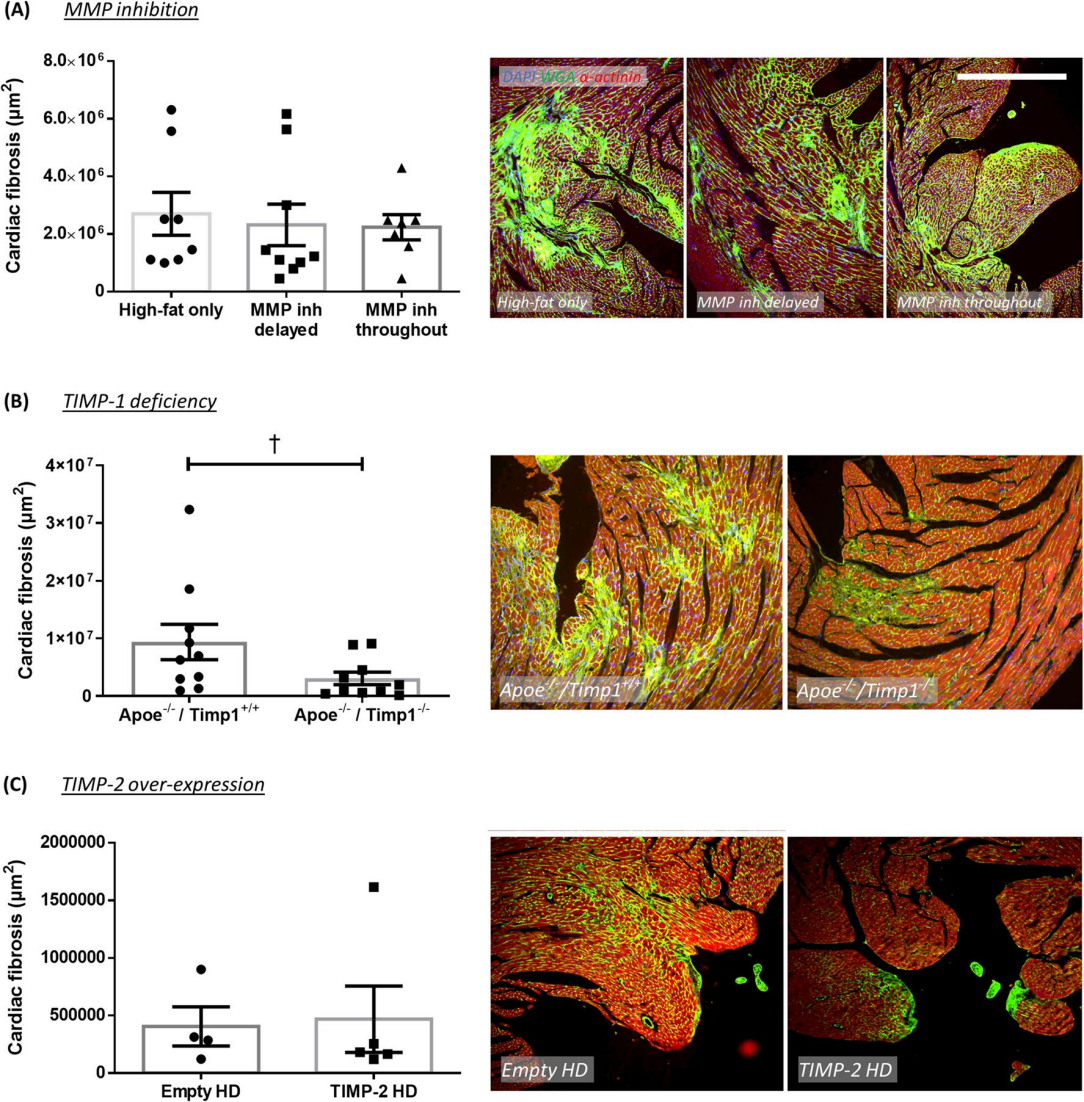
Effect of MMP inhibition and TIMP modulation on cardiac fibrosis in apolipoprotein E-deficient (Apoe^-/-^) mice. Quantification and representative images of cardiac fibrosis as assessed in WGA-stained hearts from (**A**) 36-week high-fat fed Apoe^-/-^ mice treated with or without a broad-spectrum MMP inhibitor, (**B**) 36-week high-fat fed Apoe^-/-^ mice deficient for TIMP-1 in comparison to Apoe single knockout controls, and (**C**) 18-week high-fat fed Apoe^-/-^ mice with administration of a TIMP-2 helper-dependent (HD) adenovirus or an empty HD as control. Cardiac fibrosis is presented as average fibrillar collagen content (green) in µm^2^/heart cross-section/mouse. Statistical significance is reported as †*P* < 0.05 using Mann–Whitney test. Scale bar represents 1 mm and is applicable to all panels. In A, n = 7–9/group; in B, n = 10/group; in C, n = 4–5/group.

Given the indications of MI in long-term high-fat-fed Apoe^−/−^ mice (Fig. 2) alongside the prevalence of highly stenotic coronary atherosclerosis (Figs. 2, 3, 4), cardiac fibrosis was investigated as a surrogate marker of MI, secondary to unstable coronary artery atherosclerosis. Hearts from Apoe/MMP-7 double knockout mice displayed significantly larger areas of cardiac fibrosis in comparison to strain-matched Apoe single knockout controls (3.5-fold; *P* < 0.001; Fig. 5A). Conversely, deficiency of TIMP-1 in Apoe^−/−^ mice resulted in a significantly diminished area of cardiac fibrosis compared to hearts from their respective control mice (67%; *P* < 0.05; Fig. 6B). However, MMP-9 or MMP-12 deficiency, broad spectrum MMP inhibition (delayed or throughout), or TIMP-2 overexpression had no significant effect on the extent of cardiac fibrosis (Figs. 5 and 6).

### Effect of MMP/TIMP modulation on capillary to myocyte ratio in atherosclerotic Apoe^−/−^ mice

**Figure 7:**
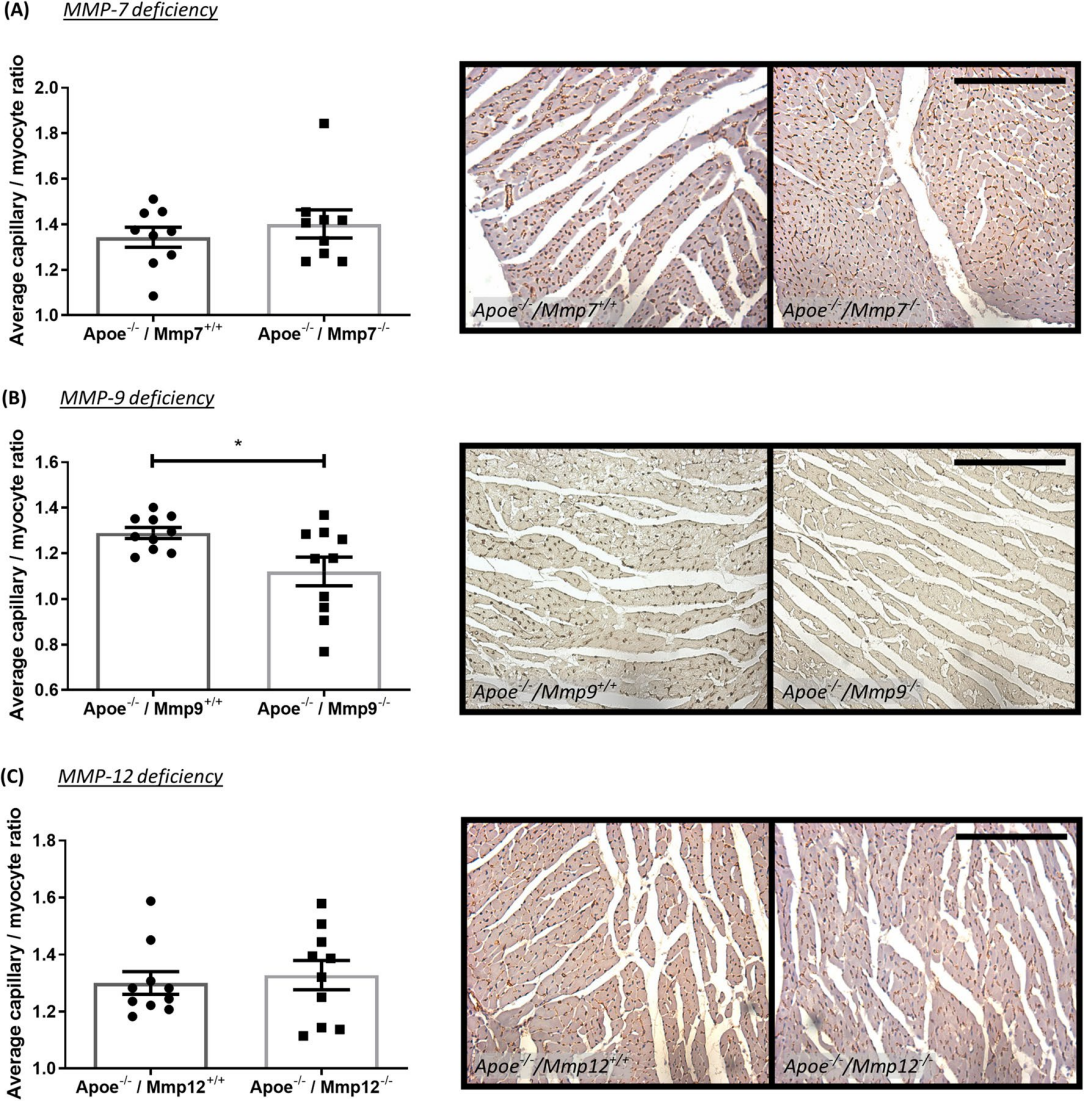
Effect of MMP modulation on capillary to myocyte ratio in apolipoprotein E-deficient (Apoe^-/-^) mice. Quantification and representative images of capillary to myocyte ratio as assessed in isolectin B4-immunohistochemically stained hearts from 36-week high-fat fed Apoe^-/-^ mice deficient for (**A**) MMP-7, (**B**) MMP-9, and (**C**) MMP-12, in comparison to their respective Apoe single knockout controls. Capillary to myocyte ratio is presented as average/heart cross-section/mouse. Statistical significance is reported as *P < 0.05 using unpaired Students t-test. Scale bar represents 1 mm and is applicable to all panels. In A, n = 9/group; in B and C, n = 10/group.

**Figure 8:**
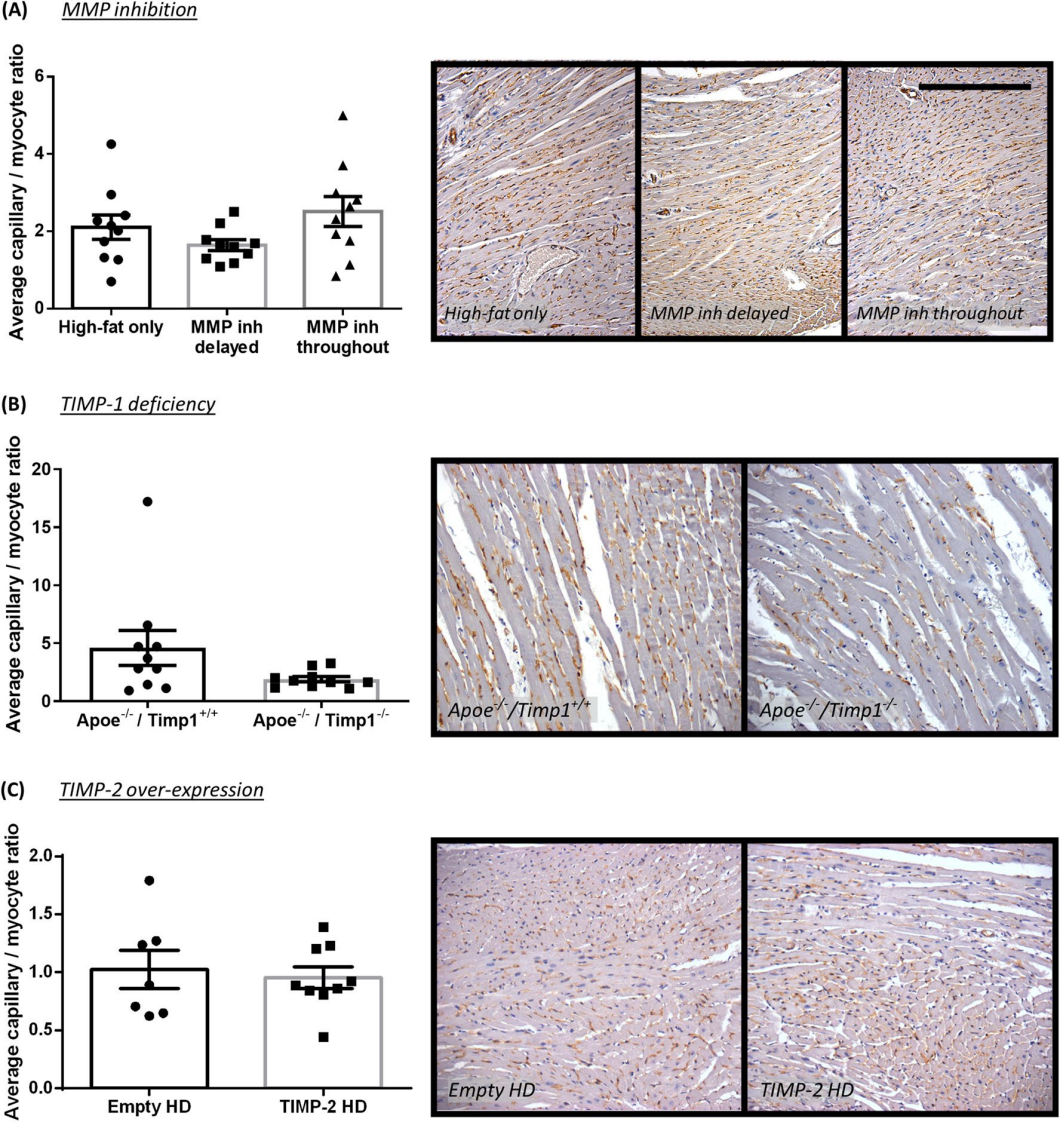
Effect of MMP inhibition and TIMP modulation on capillary to myocyte ratio in atherosclerotic apolipoprotein E-deficient (Apoe^-/-^) mice. Quantification and representative images of capillary to myocyte ratio as assessed in isolectin B4-immunohistochemically stained hearts from (**A**) 36-week high-fat fed Apoe^-/-^ mice treated with or without a broad-spectrum MMP inhibitor, (**B**) 36-week high-fat fed Apoe^-/-^ mice deficient for TIMP-1 in comparison to Apoe single knockout controls, and (**C**) 18-week high-fat fed Apoe^-/-^ mice with administration of a TIMP-2 helper-dependent (HD) adenovirus or an empty HD as control. Capillary to myocyte ratio is presented as average/heart cross-section/mouse. Scale bar represents 1 mm and is applicable to all panels. In A and B, n = 10/group; in C, n = 7–9/group.

In order to further investigate the role of MMPs and TIMPs to atherosclerosis-induced post-MI cardiac remodelling, the capillary to myocyte ratio was investigated as a surrogate marker of cardiac remodelling in atherosclerotic Apoe^−/−^ mice with signs of MI and cardiac fibrosis. MMP-7 or MMP-12 deficiency, and MMP inhibition (delayed or throughout) had no effect on the capillary to myocyte ratio (Figs. 7 and 8) or on the absolute number of capillaries or myocytes (Supplemental Fig. 2). However, MMP-9 deficiency resulted in a modest, yet statistically significant, reduction in capillary to myocyte ratio (13%; *P* < 0.05; Fig. 7B). This reduction in the capillary to myocyte ratio was associated with an increase in myocyte number (1.3-fold; *P* < 0.01; Supplementary Fig. 2b). Conversely, hearts from Apoe/TIMP-1 double knockout mice had significantly less capillaries (33%; *P* < 0.05; Supplemental Fig. 2e) but comparable number of myocytes, although this failed to translate into a difference in capillary to myocyte ratio (Fig. 8B). TIMP-2 overexpression had no effect on capillary and myocyte numbers (Supplemental Fig. 1f), or their ratio (Fig. 8C).

## Discussion

An imbalance between MMP and TIMP levels facilitates ECM remodelling during the development, progression, and rupture of atherosclerotic plaques, and during subsequent cardiac fibrosis and remodelling secondary to MI^1,2^. Consequently, this study utilised genetic deficiency of select MMPs/TIMPs alongside strategies to retard MMP activity to assess the contribution of specific MMPs and TIMPs to the development of coronary atherosclerosis and related cardiac fibrosis and remodelling in hypercholesterolaemic Apoe^−/−^ mice. The principal findings indicate divergent roles for MMPs and TIMPs in the development of coronary atherosclerosis and associated cardiac fibrosis/remodelling, suggesting that different MMPs/TIMPs can have opposing roles and act beneficially or detrimentally during atherosclerosis and post-MI remodelling. Such divergency may underly the observed ineffectiveness of broad spectrum MMP inhibition on both pathologies. In line with numerous murine experimental findings and human clinical trials, which demonstrated no beneficial effects of broad-spectrum MMP inhibition on atherosclerotic plaque formation and progression^24^, or adverse post-MI remodelling and subsequent heart failure prevention^25^, long-term or delayed administration of a non-selective MMP inhibitor (RS-130830) failed to exert beneficial changes in high-fat fed Apoe^−/−^ mice. Moreover, in studies where beneficial effects of non-selective MMP inhibition on cardiac function and remodelling were observed, opposing deleterious actions were reported when the inhibitors were administered at different stages of disease (pre- or post-MI) or time of follow-up^25,26^. As such, identifying appropriate select culprit MMPs for therapeutic targeting may provide efficacious alongside ascertaining the most effective therapeutic window to avoid adverse events.

### MMP-7

We demonstrate that Apoe/MMP-7 double knockout mice presented with reduced atherosclerotic burden and increased cardiac fibrosis, alongside a heightened incidence of sudden death after 36-weeks of high-fat feeding, suggesting that MMP-7 has an adverse role in atherosclerosis, whereas, after an MI, MMP-7 activity prevents aberrant fibrosis. The indicated adverse role for MMP-7 in atherosclerosis progression, and potential plaque vulnerability is in accordance with a previous study assessing brachiocephalic artery atherosclerosis in mice^5^, alongside reports of increased macrophage foam cell MMP-7 expression in rupture-prone human carotid plaques^27^, and an association in plasma MMP-7 levels with plaque phenotype and mortality rates^28^. Mechanistically, MMP-7 can cleave N-cadherin from the cell surface of VSMCs, promoting their apoptosis^29^, which may clarify the increased plaque VSMC content observed in MMP-7 knockout mice^5^, and support the association of increased MMP-7 levels with VSMC apoptosis and fibrous cap thinning in murine and human plaques^27,29^.

Conversely, our findings imply MMP-7 imparts a protective role during the post-MI cardiac remodelling process. During post-MI remodelling, the healing scar tissue predominantly consists of cardiac fibroblasts and marginating macrophages within the infarct border region, with macrophages and cardiomyocytes (within remote regions) both shown to express comparable MMP-7 levels after MI^30^. However, in an LAD coronary artery ligation mouse model, MMP-7 deficiency was associated with increased macrophage accumulation after MI^30^; although effects on invasion and regression were not explored to yield mechanistic insight into the heightened macrophage accumulation.

Unfortunately, although the aforementioned study demonstrated that MMP-7 deficiency had no effect on infarct size, it did not directly assess cardiac fibrosis^30^. In the context of fibrosis, despite its multiple ECM substrates MMP-7 has been characterised as pathologic mediator of fibrosis expansion in the lungs^31^ and liver^32,33^. However, in support of our observations, administration of a selective MMP-7 inhibitor suppressed cardiac fibrosis in a mouse model of uraemia-induced cardiac remodelling^34^. We therefore propose that macrophage accumulation and their accompanying release of MMP-7 within advanced plaques and during the early phase of post-MI remodelling perturb mesenchymal cell growth and survival (VSMCs in plaques and cardiac fibroblasts within the myocardium), and their associated release of ECM proteins, such as collagens.

Therefore, we speculate that while inhibiting MMP-7 activity may maintain the VSMC- and ECM-rich fibrous cap of plaques – protecting them from ensuing rupture, targeting MMP-7 during the early stages of post-MI cardiac remodelling would increase cardiac fibrosis due to loss of ECM/collagen turnover, predisposing to heart failure. As such, therapeutic strategies to blunt MMP-7 activity in unstable plaques or increase its expression after an MI would require approaches permitting localised delivery and action to limit the potential adverse effects.

### MMP-9

In this study, Apoe/MMP-9 double knockout mice had no significant changes in terms of occurrence of sudden death or coronary artery atherosclerosis. The previously reported contribution of MMP-9 to the development of atherosclerotic lesions in hypercholesterolaemic mouse models varies, depending on the vascular bed assessed. An adverse role was attributed to MMP-9 in the formation of aortic lesions^35^, while it was deemed protective against plaque development within the brachiocephalic artery^5^, through enhanced plaque stability^5,36^ via promotion of VSMC proliferation, migration, and intimal growth^37–39^.

Regarding cardiac remodelling, Apoe/MMP-9 double knockout mice displayed similar cardiac fibrosis to their relevant control mice, with a modest reduction in the capillary to myocyte ratio. A similar lack of effect on myocardial collagen content was reported in two previous studies using the LAD coronary artery ligation-induced MI mouse model alongside MMP-9 deficiency^40^ or transgenic overexpression of MMP-9^41^. However, other murine studies deploying global deficiency or macrophage-restricted overexpression of MMP-9 within the LAD ligation-induced MI model have associated MMP-9 expression with reduced cardiac fibrosis post-MI, alongside diminished macrophage accumulation and left ventricular enlargement, but enhanced neovascularization^42,43^. We observed no effect of MMP-9 deficiency on capillary number, with the reduction in the capillary to myocyte ratio driven through an increase in myocyte number. However, we did not explore if the increased myocyte number afforded from MMP-9 deficiency is through effects on myocyte survival or proliferation. In support of the former, autophagy promotes survival of myocytes during the early inflammatory response post-MI^44^, and MMP-9 inhibition increased autophagy in rat hearts with post-MI chronic heart failure^45^.

### MMP-12

Coronary arteries from Apoe/MMP-12 double knockout mice displayed significantly reduced atherosclerosis which was associated with a decreased incidence of sudden death over the course of thirty-six weeks of high-fat feeding. Previous studies in animal models and humans support a pro-atherosclerotic role for MMP-12. Rabbit studies utilising macrophage-specific overexpression of MMP-12 demonstrated that plaque development and progression were accelerated^46,47^. Similarly, global deletion of MMP-12 or administration of an MMP-12 specific inhibitor in Apoe-deficient mice, blunted brachiocephalic artery atherosclerotic plaque formation and progression, respectively^5,48^. Finally, macrophage foam cell expression of MMP-12 in human carotid plaques is associated with symptomatic severity and predicts future adverse cardiovascular events^49^. Our findings strengthen the call for therapeutic clinical trials targeting MMP-12 for the prevention of atherosclerosis.

Despite the protective effect on coronary artery atherosclerotic burden, MMP-12 deficiency had no effect on cardiac fibrosis or capillary to myocyte ratio after thirty-six weeks of high-fat feeding. Previous findings have shown MMP-12 levels are rapidly elevated within the hearts of mice post-MI (through LAD ligation), predominantly due to the inflammatory response, particularly neutrophils^50,51^. Within the same model, MMP-12 inhibition did not affect infarct size or mRNA expression of fibrillar collagens (Col1a1 and Col3a1), although cardiac fibrosis was not evaluated^50^.

While the contrasting roles for MMP-12 in atherosclerosis and post-MI remodelling may be ascribed to different cell sources and substrates, and chronic versus acute inflammatory responses, respectively, hypercholesterolaemia and underlying atherosclerosis may also explain the divergent observations. High-fat feeding of Apoe-deficient mice and ensuing hypercholesterolaemia is associated with sustained monocytosis, contributing to macrophage accrual within developing atherosclerotic plaques^52^. Accordingly, in our study the coronary vessels and myocardium have been exposed to heightened inflammation for months before the formation of coronary atherosclerosis and associated cardiac changes, whereas hypercholesterolaemia and monocytosis are absent within wild-type mice before LAD ligation-induced MI. This difference between our atherosclerosis-induced cardiac fibrosis model and the LAD ligation model may explain the observed cardiac-related discrepancies.

### TIMP-1

Atherosclerotic plaque burden and degree of stenosis were increased within the coronary arteries of Apoe/TIMP-1 double knockout mice, mirroring previous observations within the aortic root^53^ and consistent with the reported athero-protective effect afforded from systemic TIMP-1 overexpression in Apoe-deficient mice^54^. Yet, a comparable study revealed no beneficial effects of TIMP-1 overexpression on aortic atherosclerosis^55^. Similarly, TIMP-1 deficiency was shown to exert no change in atherosclerotic plaque size within the aortic root^56^ or brachiocephalic arteries^12^ of Apoe-deficient mice. These discrepancies suggest that TIMP-1 levels, and by inference inhibition of MMP activity, may differentially affect atherosclerotic plaque growth depending on the vascular bed and stage of atherosclerosis, with influence on plaque composition more relevant than plaque size in advanced plaques^24^.

Interestingly, the increased coronary plaque burden observed in Apoe/TIMP-1 double knockout mice was associated with a marked reduction in cardiac fibrosis. Comparable findings have been reported in the LAD ligation model 14-days post-MI^57^, implying the increased MMP activity provided from TIMP-1 deficiency promotes adverse myocardial remodelling, accelerating subsequent heart failure. Although, clinical evidence revealed elevated TIMP-1 expression in patients with extended cardiac fibrosis due to chronic pressure overload or heart failure^58,59^, which may reflect compensatory upregulation of TIMP-1 or the complex role of fibrosis post-MI. We also observed decreased capillary number within the hearts of Apoe/TIMP-1 double knockout mice, reflecting adverse myocardial wound healing due to perturbed neovascularisation. Indeed, fibroblast-derived TIMP-1 can prevent MMP-dependent cleavage and generation of anti-angiogenic collagen IV fragments^60^. Although, previous reports suggest that blocking TIMP-1 increases angiogenesis in an in vivo sponge model of angiogenesis^61^. These findings collectively suggest that TIMP-1 is protective against the development of coronary atherosclerosis, while aggravating cardiac fibrosis and affecting the remodelling process.

### TIMP-2

Studies assessing brachiocephalic artery atherosclerosis have consistently demonstrated an athero-protective role for TIMP-2^12,13^. However, TIMP-2 overexpression did not alter coronary artery plaque burden or degree of stenosis in the current study. This may be due to the reduced prevalence of stenotic plaques within this sub-study as mice were high-fat-fed for 18-weeks only, with potentially longer-term feeding required to detect an effect on plaque burden, as observed in MMP-7 and MMP-12-deficient mice.

Despite evidence demonstrating that TIMP-2 is upregulated in chronic pressure-overloaded human hearts^59^ and post-MI in the mouse LAD ligation model, our findings did not establish a link between TIMP-2 overexpression and cardiac collagen density. This lack of effect may be associated with the short duration of high-fat feeding and associated diminished coronary atherosclerosis, resulting in less adverse cardiac events as seen in the longer-term experiments. Indeed, loss of TIMP-2 has been shown to hasten adverse post-MI remodelling, as evidenced through decreased collagen content alongside greater infarct size, both attributed to increased inflammation and associated MMP-14 expression and activity^62^. Although TIMP-2 overexpression did not impart any protective effects in atherosclerosis or cardiac fibrosis, longer-term studies would be appropriate to evaluate its therapeutic potential to combat the progression of both diseases. In addition, assessing the effect of TIMP-2 deficiency in long-term high-fat fed Apoe-deficient mice may also provide useful in the pathophysiological assessment of both pathologies.

## Conclusions

This study highlights the divergent roles of specific MMPs and TIMPs in hypercholesterolaemia-induced coronary artery atherosclerotic plaque formation alongside cardiac fibrosis and remodelling, secondary to MI. Overall, the data suggest that nuanced and selective therapeutic approaches, that target specific MMPs or TIMPs during a defined therapeutic window, are necessary for treatments of both atherosclerosis and adverse post-infarction remodelling to avoid opposing deleterious effects on each pathology. The absence of adverse consequences on cardiac fibrosis with MMP-12 deficiency, supports targeting of MMP-12 for the treatment of unstable atherosclerosis. Finally, the concurrent spontaneous development of advanced coronary atherosclerosis and MI-associated cardiac fibrosis support use of the prolonged high-fat fed Apoe-deficient mouse as an alternative to the LAD occlusion model of nonperfused MI and models of reperfused MI^63^.

## Supplementary Information

Supplementary Information.

## Data Availability

The data that support the findings of this study are included within the manuscript or available from the corresponding author upon reasonable request.

## Abbreviations

APOE: Apolipoprotein E
BSA: Bovine serum albumin
ECM: Extracellular matrix
EVG: Miller’s elastin/van Gieson
HD: Helper dependent
IB4: Isolectin B4
LAD: Left anterior descending
MI: Myocardial infarction
MMP: Matrix metalloproteinase
PBS: Phosphate buffered saline
SEM: Standard error of mean
TIMP: Tissue inhibitor of metalloproteinase
VEGF: Vascular endothelial growth factor
VSMC: Vascular smooth muscle cell
WGA: Wheat germ agglutinin

## References

[1] Rienks M, Papageorgiou AP, Frangogiannis NG, Heymans S (2014). Myocardial extracellular matrix: an ever-changing and diverse entity. Circ. Res..

[2] Spinale FG, Villarreal F (2014). Targeting matrix metalloproteinases in heart disease: lessons from endogenous inhibitors. Biochem. Pharmacol..

[3] Soehnlein O, Swirski FK (2013). Hypercholesterolemia links hematopoiesis with atherosclerosis. Trends. Endocrinol. Metab..

[4] Bentzon JF, Otsuka F, Virmani R, Falk E (2014). Mechanisms of plaque formation and rupture. Circ. Res..

[5] Johnson JL, George SJ, Newby AC, Jackson CL (2005). Divergent effects of matrix metalloproteinases 3, 7, 9, and 12 on atherosclerotic plaque stability in mouse brachiocephalic arteries. Proc. Natl. Acad. Sci. U S A.

[6] Johnson JL (2014). Relationship of MMP-14 and TIMP-3 expression with macrophage activation and human atherosclerotic plaque vulnerability. Med. Inflamm..

[7] van der Laan AM, Nahrendorf M, Piek JJ (2012). Healing and adverse remodelling after acute myocardial infarction: role of the cellular immune response. Heart.

[8] Fraccarollo D, Galuppo P, Bauersachs J (2012). Novel therapeutic approaches to post-infarction remodelling. Cardiovasc. Res..

[9] Frangogiannis NG (2014). The inflammatory response in myocardial injury, repair, and remodelling. Nat. Rev. Cardiol..

[10] Kaminski AR (2020). The compendium of matrix metalloproteinase expression in the left ventricle of mice following myocardial infarction. Am. J. Physiol. Heart Circ. Physiol..

[11] Velagaleti RS (2008). Long-term trends in the incidence of heart failure after myocardial infarction. Circulation.

[12] Di Gregoli K, George SJ, Jackson CL, Newby AC, Johnson JL (2016). Differential effects of tissue inhibitor of metalloproteinase (TIMP)-1 and TIMP-2 on atherosclerosis and monocyte/macrophage invasion. Cardiovasc. Res..

[13] Johnson JL (2006). Suppression of atherosclerotic plaque progression and instability by tissue inhibitor of metalloproteinase-2: involvement of macrophage migration and apoptosis. Circulation.

[14] Plump AS (1992). Severe hypercholesterolemia and atherosclerosis in apolipoprotein E-deficient mice created by homologous recombination in ES cells. Cell.

[15] Zhang SH, Reddick RL, Piedrahita JA, Maeda N (1992). Spontaneous hypercholesterolemia and arterial lesions in mice lacking apolipoprotein E. Science.

[16] Wilson CL, Heppner KJ, Labosky PA, Hogan BLM, Matrisian LM (1997). Intestinal tumorigenesis is suppressed in mice lacking the metalloproteinase matrilysin. Proc. Natl. Acad. Sci..

[17] Vu TH (1998). MMP-9/gelatinase B is a key regulator of growth plate angiogenesis and apoptosis of hypertrophic chondrocytes. Cell.

[18] Shipley JM, Wesselschmidt RL, Kobayashi DK, Ley TJ, Shapiro SD (1996). Metalloelastase is required for macrophage-mediated proteolysis and matrix invasion in mice. Proc. Natl. Acad. Sci. USA.

[19] Soloway P, Alexander CM, Werb Z, Jaenisch R (1996). Targeted mutagenesis of TIMP-1 reveals that lung tumour invasion is influenced by TIMP-1 genotype of the tumour but not by that of the host. Oncogene.

[20] Johnson JL (2006). Effect of broad-spectrum matrix metalloproteinase inhibition on atherosclerotic plaque stability. Cardiovasc. Res..

[21] Johnson JL, Jackson CL (2001). Atherosclerotic plaque rupture in the apolipoprotein E knockout mouse. Atherosclerosis.

[22] Williams H, Johnson JL, Carson KGS, Jackson CL (2002). Characteristics of intact and ruptured atherosclerotic plaques in brachiocephalic arteries of apolipoprotein E knockout mice. Arterioscler. Thromb. Vasc. Biol..

[23] Johnson J (2005). Plaque rupture after short periods of fat-feeding in the apolipoprotein E knockout mouse: model characterisation, and effects of pravastatin treatment. Circulation.

[24] Johnson JL (2017). Metalloproteinases in atherosclerosis. Eur. J. Pharmacol..

[25] DeLeon-Pennell KY, Meschiari CA, Jung M, Lindsey ML (2017). Matrix metalloproteinases in myocardial infarction and heart failure. Prog. Mol. Biol. Transl. Sci..

[26] Yarbrough WM (2003). Selective targeting and timing of matrix metalloproteinase inhibition in post-myocardial infarction remodeling. Circulation.

[27] Halpert I (1996). Matrilysin is expressed by lipid-laden macrophages at sites of potential rupture in atherosclerotic lesions and localizes to areas of versican deposition, a proteoglycan substrate for the enzyme. Proc. Natl. Acad. Sci. USA.

[28] Abbas A (2014). Matrix metalloproteinase 7 is associated with symptomatic lesions and adverse events in patients with carotid atherosclerosis. PLoS ONE.

[29] Williams H, Johnson JL, Jackson CL, White SJ, George SJ (2010). MMP-7 mediates cleavage of N-cadherin and promotes smooth muscle cell apoptosis. Cardiovasc. Res..

[30] Lindsey ML (2006). Matrix metalloproteinase-7 affects connexin-43 levels, electrical conduction, and survival after myocardial infarction. Circulation.

[31] Zuo F (2002). Gene expression analysis reveals matrilysin as a key regulator of pulmonary fibrosis in mice and humans. Proc. Natl. Acad. Sci. U S A.

[32] Huang CC (2005). Matrilysin (MMP-7) is a major matrix metalloproteinase upregulated in biliary atresia-associated liver fibrosis. Mod. Pathol..

[33] Lichtinghagen R (2001). Matrix metalloproteinase (MMP)-2, MMP-7, and tissue inhibitor of metalloproteinase-1 are closely related to the fibroproliferative process in the liver during chronic hepatitis C. J. Hepatol..

[34] Yang Y (2020). Matrix metalloproteinase-7 in platelet-activated macrophages accounts for cardiac remodeling in uremic mice. Basic Res. Cardiol..

[35] Luttun A (2004). Loss of matrix metalloproteinase-9 or matrix metalloproteinase-12 protects apolipoprotein E-deficient mice against atherosclerotic media destruction but differentially affects plaque growth. Circulation.

[36] Johnson JL, Dwivedi A, Somerville M, George SJ, Newby AC (2011). Matrix metalloproteinase (MMP)-3 activates MMP-9 mediated vascular smooth muscle cell migration and neointima formation in mice. Arterioscler Thromb. Vasc. Biol..

[37] Cho A, Reidy MA (2002). Matrix metalloproteinase-9 is necessary for the regulation of smooth muscle cell replication and migration after arterial injury. Circ. Res..

[38] Choi ET (2005). Matrix metalloproteinase-9 modulation by resident arterial cells is responsible for injury-induced accelerated atherosclerotic plaque development in apolipoprotein E-deficient mice. Arterioscler Thromb. Vasc. Biol..

[39] Johnson C, Galis ZS (2004). Matrix metalloproteinase-2 and-9 differentially regulate smooth muscle cell migration and cell-mediated collagen organization. Arterioscler Thromb. Vasc. Biol..

[40] Ramirez TA (2014). Aliskiren and valsartan mediate left ventricular remodeling post-myocardial infarction in mice through MMP-9 effects. J. Mol. Cell. Cardiol..

[41] Zamilpa R (2012). Transgenic overexpression of matrix metalloproteinase-9 in macrophages attenuates the inflammatory response and improves left ventricular function post-myocardial infarction. J. Mol. Cell. Cardiol..

[42] Iyer RP, Jung M, Lindsey ML (2016). MMP-9 signaling in the left ventricle following myocardial infarction. Am. J. Physiol. Heart Circ. Physiol..

[43] Ducharme A (2000). Targeted deletion of matrix metalloproteinase-9 attenuates left ventricular enlargement and collagen accumulation after experimental myocardial infarction. J. Clin. Invest..

[44] Wang X, Guo Z, Ding Z, Mehta JL (2018). Inflammation, autophagy, and apoptosis after myocardial infarction. J. Am. Heart Assoc..

[45] Nandi SS (2020). MMP9 inhibition increases autophagic flux in chronic heart failure. Am. J. Physiol. Heart Circ. Physiol..

[46] Yamada S (2008). Matrix metalloproteinase 12 accelerates the initiation of atherosclerosis and stimulates the progression of fatty streaks to fibrous plaques in transgenic rabbits. Am. J. Pathol..

[47] Liang J (2006). Macrophage metalloelastase accelerates the progression of atherosclerosis in transgenic rabbits. Circulation.

[48] Johnson JL (2011). A selective matrix metalloproteinase-12 inhibitor retards atherosclerotic plaque development in apolipoprotein E-knockout mice. Arterioscler Thromb. Vasc. Biol..

[49] Scholtes VPW (2012). Carotid atherosclerotic plaque matrix metalloproteinase-12–positive macrophage subpopulation predicts adverse outcome after endarterectomy. JAHA.

[50] Iyer RP (2015). Early matrix metalloproteinase-12 inhibition worsens post-myocardial infarction cardiac dysfunction by delaying inflammation resolution. Int. J. Cardiol..

[51] Kubota A, Suto A, Suzuki K, Kobayashi Y, Nakajima H (2019). Matrix metalloproteinase-12 produced by Ly6C low macrophages prolongs the survival after myocardial infarction by preventing neutrophil influx. J. Mol. Cell. Cardiol..

[52] Swirski FK (2007). Ly-6Chi monocytes dominate hypercholesterolemia-associated monocytosis and give rise to macrophages in atheromata. J. Clin. Invest..

[53] Silence J, Collen D, Lijnen HR (2002). Reduced atherosclerotic plaque but enhanced aneurysm formation in mice with inactivation of the tissue inhibitor of metalloproteinase-1 (TIMP-1) gene. Circ. Res..

[54] Rouis M (1999). Adenovirus-mediated overexpression of tissue inhibitor of metalloproteinase-1 reduces atherosclerotic lesions in apolipoprotein E-deficient mice. Circulation.

[55] Cuaz-Perolin C (2006). Apolipoprotein E knockout mice over-expressing human tissue inhibitor of metalloproteinase 1 are protected against aneurysm formation but not against atherosclerotic plaque development. J. Vasc. Res..

[56] Lemaître V, Soloway PD, D'Armiento J (2003). Increased medial degradation with pseudo-aneurysm formation in apolipoprotein E-knockout mice deficient in tissue inhibitor of metalloproteinases-1. Circulation.

[57] Ikonomidis JS (2005). Accelerated LV remodeling after myocardial infarction in TIMP-1-deficient mice: effects of exogenous MMP inhibition. Am. J. Physiol. Heart Circ. Physiol..

[58] Wu CK, Su MM, Wu YF, Hwang JJ, Lin LY (2018). Combination of plasma biomarkers and clinical data for the detection of myocardial fibrosis or aggravation of heart failure symptoms in heart failure with preserved ejection fraction patients. J. Clin. Med..

[59] Heymans S (2005). Increased cardiac expression of tissue inhibitor of metalloproteinase-1 and tissue inhibitor of metalloproteinase-2 is related to cardiac fibrosis and dysfunction in the chronic pressure-overloaded human heart. Circulation.

[60] Liu H, Chen B, Lilly B (2008). Fibroblasts potentiate blood vessel formation partially through secreted factor TIMP-1. Angiogenesis.

[61] Reed MJ, Koike T, Sadoun E, Sage EH, Puolakkainen P (2003). Inhibition of TIMP1 enhances angiogenesis in vivo and cell migration in vitro. Microvasc. Res..

[62] Kandalam V (2010). TIMP2 deficiency accelerates adverse post-myocardial infarction remodeling because of enhanced MT1-MMP activity despite lack of MMP2 activation. Circ. Res..

[63] Lindsey ML (2021). Reperfused vs. nonreperfused myocardial infarction: when to use which model. Am. J. Physiol. Heart Circ. Physiol..

