# Supplementary material for: Disparate effects of MMP and TIMP modulation on coronary atherosclerosis and associated myocardial fibrosis
Source: Sci Rep. 2021 Nov 30;11:23081. doi: 10.1038/s41598-021-02508-4 (PMC8632906; doi:10.1038/s41598-021-02508-4)
Supplement: Supplementary file 1 — Supplementary Information. [file 41598_2021_2508_MOESM1_ESM.pdf]

Table 1: Effect of MMP/TIMP modulation on coronary atherosclerosis and myocardial composition in apolipoprotein E-deficient (Apoe$^{-/-}$) mice

	Apoe$^{-/-}$ / MMP-7$^{-/-}$	Apoe$^{-/-}$ / MMP-9$^{-/-}$	Apoe$^{-/-}$ / MMP-12$^{-/-}$	MMP inh delayed	MMP inh throughout	Apoe$^{-/-}$ / TIMP-1$^{-/-}$	TIMP-2 HD
**Sudden death**	↑	ns	↓	ns	ns	ns	↓
**Atherosclerotic burden**	↓	ns	↓	ns	ns	↑	ns
**Atherosclerotic plaque stenosis**	ns	ns	ns	ns	ns	↑	ns
**Fibrosis**	↑	ns	ns	ns	ns	↓	ns
**Capillary to myocyte ratio**	ns	↓	ns	ns	ns	ns	ns
**Capillary number**	ns	ns	ns	ns	ns	↓	ns
**Myocyte number**	ns	↑	ns	ns	ns	ns	ns

Supplementary Figure 1: Effect of MMP/TIMP modulation on coronary artery atherosclerotic plaque stenosis in apolipoprotein E-deficient (Apoe$^{-/-}$) mice

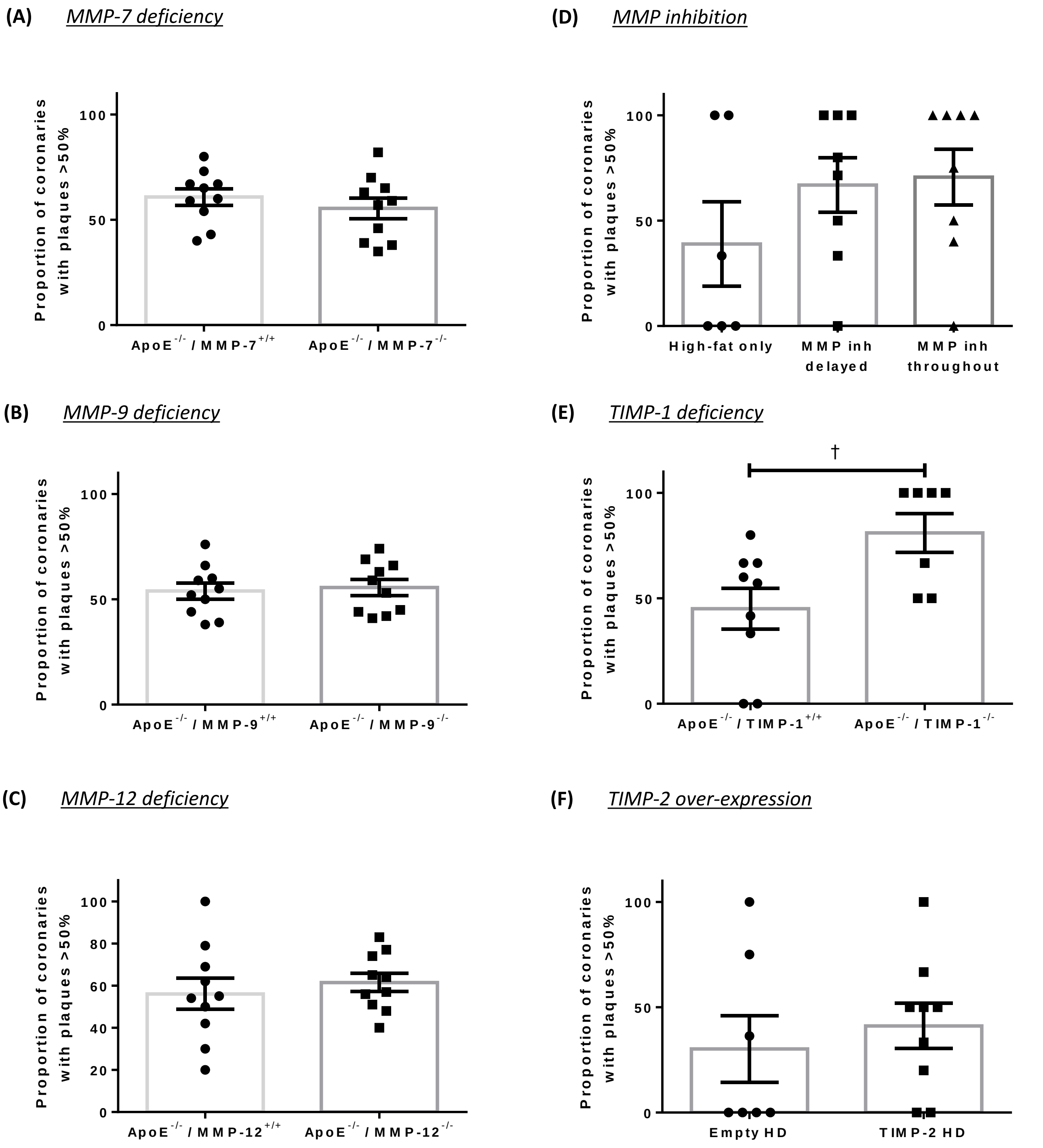

Supplementary Figure 1: Effect of MMP/TIMP modulation on coronary artery atherosclerotic plaque stenosis in apolipoprotein E-deficient (Apoe$^{-/-}$) mice

In atherosclerotic Apoe$^{-/-}$ mice (E) TIMP-1 deficiency was associated with increased coronary artery plaque stenosis, represented as proportion of coronary arteries occupying more than 50% of the lumen. There were no significant differences between (A) MMP-7, (B) MMP-9, or (C) MMP-12 double knockout mice, (D) MMP (delayed or throughout) inhibition, and (F) TIMP-2 overexpression and their respective controls. Statistical significance is reported as *$p<0.05$, **$p<0.01$, ***$p<0.001$, and †$p<0.05$ is used when non-parametric test was used. In A-E, n=10/group; in F, n=7-9/group.

Supplementary Figure 2: Effect of MMP/TIMP modulation on capillary and myocyte numbers in atherosclerotic apolipoprotein E-deficient (Apoe$^{-/-}$) mice

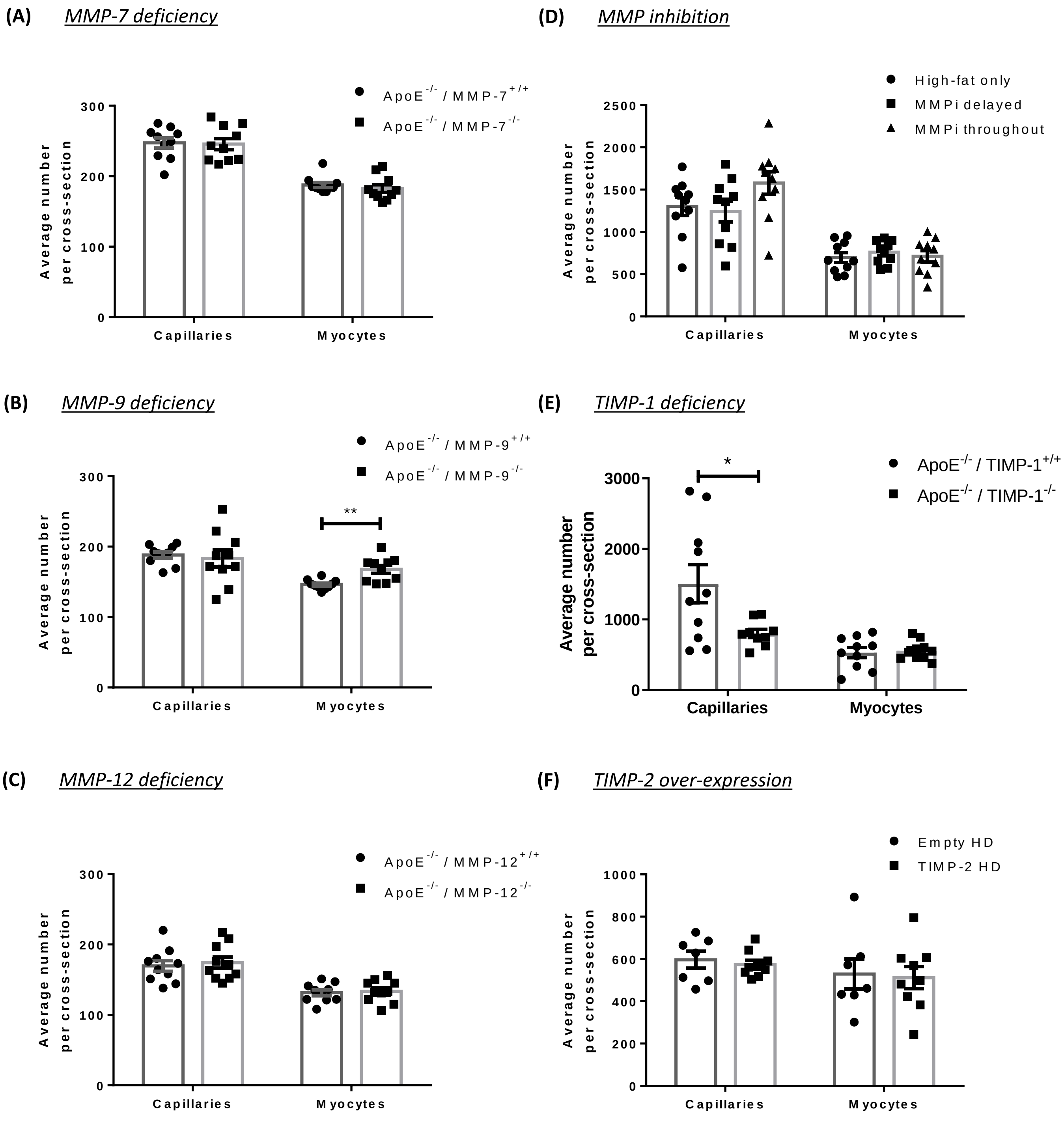

Supplementary Figure 2: Effect of MMP/TIMP modulation on capillary and myocyte numbers in atherosclerotic apolipoprotein E-deficient (Apoe$^{-/-}$) mice.

In atherosclerotic Apoe$^{-/-}$ mice (B) MMP-9 deficiency was associated with an increased number of myocytes without any change in absolute number of capillaries. (E) TIMP-1 deficiency significantly reduced the number of capillaries without any change in the number of myocytes. There were no significant changes in the number of capillaries or myocytes between (A) MMP-7 or (C) MMP-12 double knockout mice, (D) MMP (delayed or throughout) inhibition, and (F) TIMP-2 overexpression and their respective controls. Statistical significance is reported as *$p<0.05$, **$p<0.01$, ***$p<0.001$, and †$p<0.05$ is used when non-parametric test was used. In A-E, n=10/group; in F, n=7-9/group.
